# A unified dataset for the city-scale traffic assignment model in 20 U.S. cities

**DOI:** 10.1038/s41597-024-03149-8

**Published:** 2024-03-29

**Authors:** Xiaotong Xu, Zhenjie Zheng, Zijian Hu, Kairui Feng, Wei Ma

**Affiliations:** 1 Department of Civil and Environmental Engineering, The Hong Kong Polytechnic University, Hong Kong, 999077 China; 2 The Department of Civil and Environmental Engineering, Princeton University, Princeton, 08544 USA; 3 The Hong Kong Polytechnic University Shenzhen Research Institute, Shenzhen, Guangdong 518000 China

**Keywords:** Engineering, Engineering

## Abstract

City-scale traffic data, such as traffic flow, speed, and density on every road segment, are the foundation of modern urban research. However, accessing such data on a city scale is challenging due to the limited number of sensors and privacy concerns. Consequently, most of the existing traffic datasets are typically limited to small, specific urban areas with incomplete data types, hindering the research in urban studies, such as transportation, environment, and energy fields. It still lacks a city-scale traffic dataset with comprehensive data types and satisfactory quality that can be publicly available across cities. To address this issue, we propose a unified approach for producing city-scale traffic data using the classic traffic assignment model in transportation studies. Specifically, the inputs of our approach are sourced from open public databases, including road networks, traffic demand, and travel time. Then the approach outputs comprehensive and validated citywide traffic data on the entire road network. In this study, we apply the proposed approach to 20 cities in the United States, achieving an average correlation coefficient of 0.79 in average travel time and an average relative error of 5.16% and 10.47% in average travel speed when compared with the real-world data.

## Background & Summary

City-scale traffic data, including traffic flow, speed, and density on every road segment of the entire road network, are foundational inputs and building blocks for modern urban research. These traffic datasets offer an overview of urban mobility, facilitating a better understanding of traffic conditions and travelers’ behaviors in a city. Utilizing the city-scale traffic data, policymakers could develop appropriate transport policies and strategies to mitigate traffic congestion^1,2^. Additionally, the traffic data can also be used to evaluate the noise and air pollution caused by vehicles in urban areas^3–5^, which are important in enhancing public health and environmental conditions^6–8^. Furthermore, it assists in formulating energy-efficient traffic management and control strategies that can substantially reduce energy consumption^9–11^. In view of this, it is of great importance to produce and publish open-access traffic datasets on a city scale to support related studies in interdisciplinary research.

However, it is challenging to directly collect the traffic data on every road segment on the entire road network. This is because the traffic data are typically collected from various traffic sensors (e.g., loop detectors, CCTV cameras), which are usually insufficient to cover the entire network due to the associated high installation and maintenance costs. For instance, there are over 30,000 links on the road network of Hong Kong, but less than 10% of the links (i.e., 2,800) are equipped with volume detectors^12^. Moreover, data missing or data measurement errors are inevitable problems due to various factors such as sensor failures, software malfunctions, and weak communication signal transmission^13,14^. For example, existing studies indicate that approximately 30% of the freeway sensors in California Performance Measurement System (PeMS: https://pems.dot.ca.gov/) are not working properly, resulting in data missing^15,16^. More importantly, directly observing the traffic conditions may not be sufficient since the underlying mechanism of the traffic dynamics is not reflected. For example, a reduction in traffic speed indicates congestion, while it is still not clear how the congestion is formed^17^.

To address the above challenges, many urban planning or transport departments utilize traffic modeling techniques to estimate the city-scale traffic data in a generative manner. Specifically, the traffic assignment model^18^, which is a mature model that has been studied extensively in the transportation field, is adopted to estimate the city-scale traffic states. The input of the traffic assignment model only includes the Origin-Destination (OD) demand information and network structure, both of which are public and openly available. Then, the model outputs the city-scale traffic dataset. Traffic assignment models utilize OD data to predict traffic flow and route choices for individual travelers, relying on either predefined or data-driven behavioral models. By modeling the interactions between travelers’ behaviors and traffic congestion, the traffic assignment model searches for the equilibrium condition that mimics real-world traffic conditions. Traffic assignment models can often serve as the primary tool for local governments to assess the potential impact of changes in land use or road network expansions on both local and global traffic conditions. These models are indispensable because they inherently focus on optimizing travel decisions for local residents, aligning with their individual preferences. This capability enables the model to predict changes in agent-level behavior in situations that may not be fully reflected in the available data. Moreover, traffic assignment models demonstrate robust predictive capabilities for estimating future traffic conditions. For example, Metropolitan Planning Organizations (MPOs) in urban areas of the United States would utilize travel survey data, such as the National Household Travel Survey (NHTS: https://nhts.ornl.gov/), to produce traffic data for each local urban area that represent residents’ travel patterns^19^. However, these traffic assignment models and data are usually maintained by public agencies and generally not available to most researchers or the public due to difficulties in information sharing or privacy concerns^20,21^. Furthermore, the data used in traffic assignment models are under the ownership of various institutions and lack standardization in terms of their structures, granularity, and output formats. As a result, the data are restricted to a few researchers and it is challenging to access the necessary data for traffic assignment models across cities from official sources. Given the above, there is still a notable absence of city-scale traffic datasets that include multiple major cities within one geographic and cultural region, adhere to consistent standards, collect and validate information on a uniform scale, provide comprehensive data types, and meet high-quality standards for public availability.

Although there are a few publicly available datasets^22,23^ concerning urban areas (see Table 1), the reliability and completeness of these datasets limit their applications across broader urban studies, especially in fields like energy, environment, and public health^24,25^. The limitations come from the following aspects: First, the existing traffic datasets typically cover some important traffic segments for a single city rather than a city-scale traffic dataset for multiple cities. Second, these current datasets often lack the necessary input, including road network data and corresponding OD data, directly usable for traffic assignment models. Third, these datasets often suffer from incomplete data types and lack of timely updating, resulting in limited convenience when utilizing them. In other words, these datasets are often collected by different researchers or volunteers several years ago, leading to a lack of uniformity in the data types and formats, as well as infrequent updates and maintenance. Fourth, these datasets frequently lack comprehensive validation across multiple variables or fail to offer adequate tools for predicting traffic features from behavioral data. For example, a dataset that includes OD numbers may result in unrealistic traffic flow predictions when attempting to utilize a traffic assignment model. In light of these mentioned facts, currently, there is no unified and well-validated traffic dataset available for multiple cities that covers the entire urban road network at a citywide scale, which hinders the feasibility of conducting comprehensive urban studies across cities to unearth novel discoveries.

**Table 1:** Comparison between existing open public traffic datasets and our dataset.

Dataset	Cross-validation	City-scale	Multi-city	Traffic Assignment Model	Data Types		
					Flow	Density	Speed
Transportation Networks^49^			✓	✓	✓	✓	✓
UTD19^50^	✓		✓		✓	✓	✓
PeMS^51^			✓		✓	✓	✓
Road Traffic Statistics^52^			✓		✓		
Traffic Volumes AADT^53^			✓		✓		
Traffic Volume Counts^54^					✓	✓	✓
Traffic Data of Strategic/Major Roads^55^					✓	✓	✓
Vehicle Trajectory Data^56,57^		✓			✓		
Traffic Flow Data^58,59^		✓			✓		✓
Our dataset^46^	✓	✓	✓	✓	✓	✓	✓

To facilitate convenient access to citywide traffic assignment models and data for researchers from different domains besides transportation fields, this study provides a unified traffic dataset for traffic assignment models in 20 representative U.S. cities, with populations ranging from 0.3 million to over 8.8 million. Specifically, we first obtain the input of the model by fusing multiple open public data sources, including OpenStreetMap, The Longitudinal Employer-Household Dynamics Origin-Destination Employment Statistics (LODES), Waze, and TomTom. Then, we employ a grid-search method to fine-tune the parameters and generate the final traffic dataset for each city. The real world’s average travel time and traffic speed serve as validation criteria to ensure a reliable and effective traffic dataset for multiple cities. The validation results demonstrate that our approach can successfully produce the dataset with an average correlation coefficient of 0.79 for average travel time and an average error of 5.16% and 10.47% for average travel speed between real-world data and our data. Finally, we upload the validated traffic dataset and the code used in this study to a public repository.

**Fig. 1:**
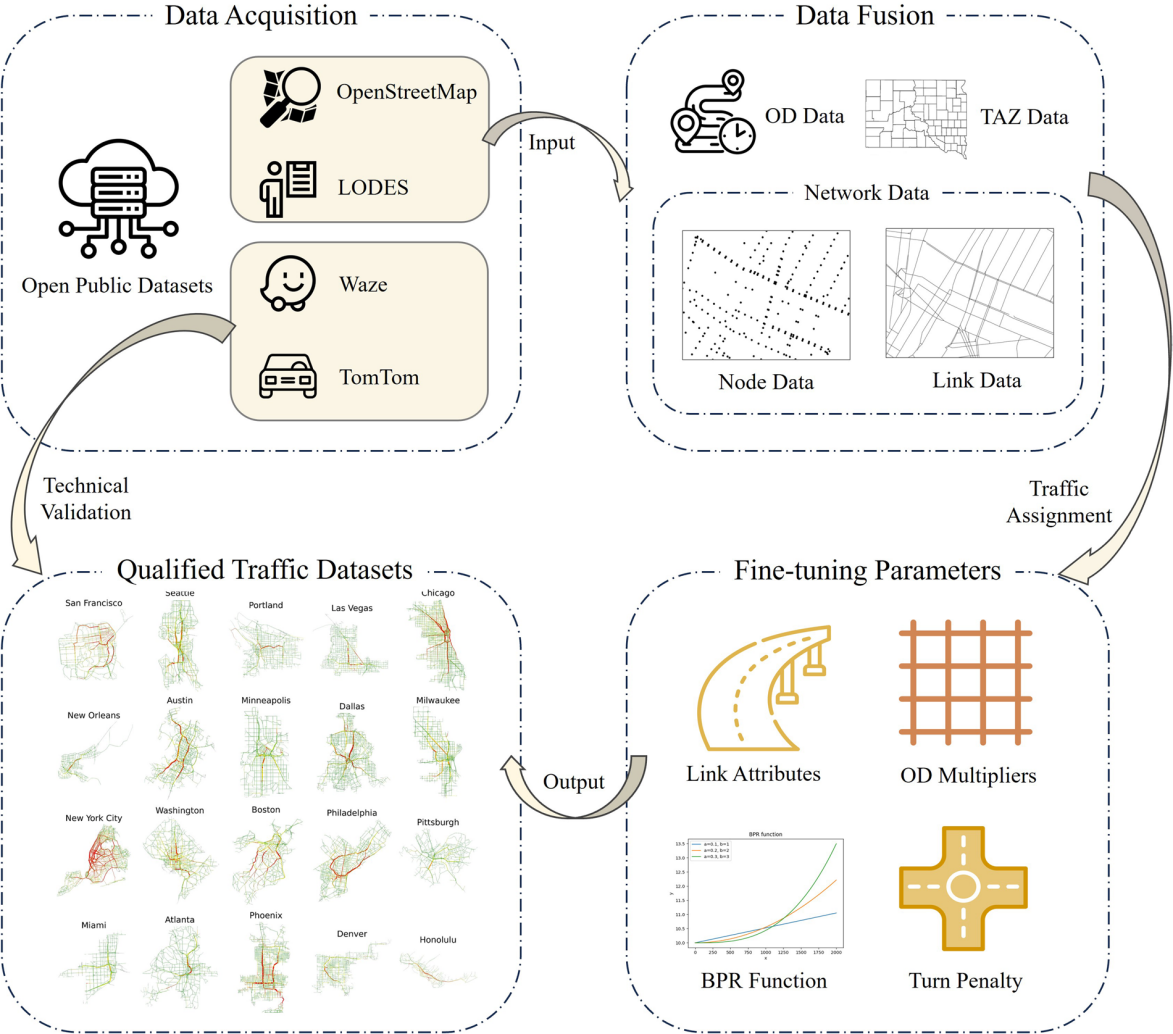
The workflow of obtaining unified and validated traffic datasets from multi-source open public datasets.

To sum up, we utilize the static traffic assignment model, leveraging annually aggregated statistical data and open public data sources, to offer a city-scale traffic dataset for macroscopic urban research. It is worth noting that the approach provided in this study can also be applied to other cities. A comprehensive workflow of processing multi-source open public datasets to acquire this dataset is provided in Fig. 1.

## Methods

Creating a unified traffic dataset in multiple cities involves four main procedures: (1) the identification of representative cities; (2) the acquisition of corresponding input data from multi-source open public datasets; (3) the fusion of the obtained data; and (4) the implementation of traffic assignment, along with parameters calibration. The main procedures are illustrated accordingly below.

### Identification of representative cities

In this study, we select a total of 20 representative cities in the United States and generate corresponding traffic datasets using the proposed approach. To ensure diversity and exemplarity among the selected cities, we primarily consider factors such as geographic location, urban scale, topography, and traffic conditions during the commute. Our selection includes a range of cities, including megacities like New York City, as well as several large cities such as Chicago and Philadelphia. We also included smaller but equally representative cities such as Honolulu. The topography of these cities also varies widely. For example, New York and San Francisco are separated by several rivers and rely on critical bridges and tunnels for commuting, while Las Vegas and Phoenix have relatively flat and continuous terrain, with surface transportation playing a predominant role.

**Fig. 2:**
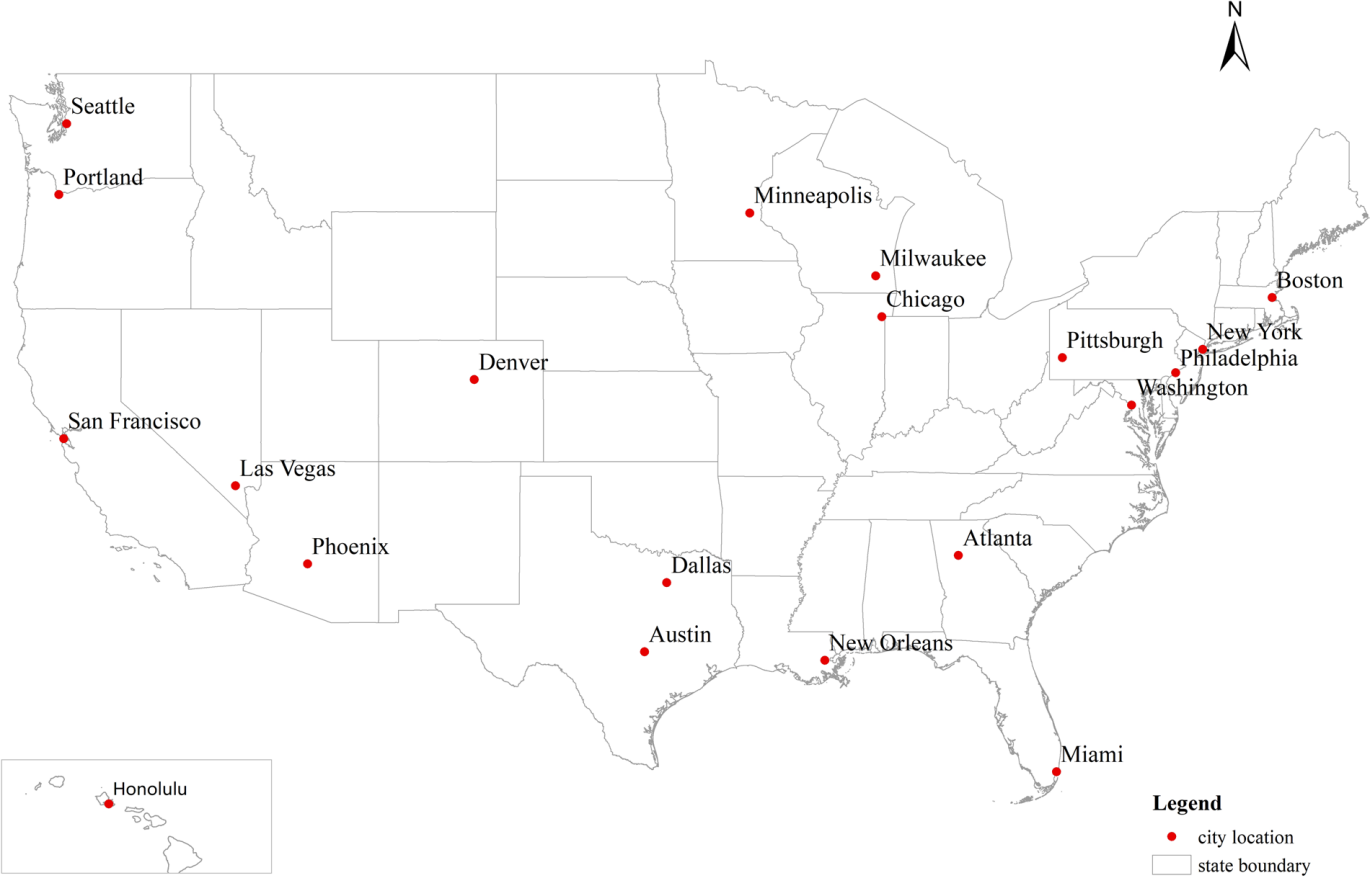
The geospatial distribution of 20 representative U.S. cities.

Basic information of the 20 representative cities in the United States is given in Table 2. The population and land area data in the year 2020 are sourced from the U.S. Census Bureau (https://www.census.gov/) while the congestion ranking information in the year 2022 is from TomTom (https://www.tomtom.com/traffic-index/ranking/). Their geospatial distribution is shown in Fig. 2.

**Table 2:** Basic information on 20 representative U.S. cities.

No.	City	State	Time Zone	Census 2020	Land Area (*km*^2^)	Population Density	Congestion Ranking
1	San Francisco	California	Pacific Time	873,965	121.5	7,195	3
2	Seattle	Washington		737,015	217.0	3,396	7
3	Portland	Oregon		652,503	345.8	1,887	16
4	Las Vegas	Nevada		641,903	367.3	1,748	25
5	Chicago	Illinois	Central Time	2,746,388	589.7	4,657	5
6	New Orleans	Louisiana		383,997	439.0	875	19
7	Austin	Texas		961,855	828.5	1,161	20
8	Minneapolis	Minnesota		429,954	139.9	3,074	27
9	Dallas	Texas		1,304,379	879.6	1,483	33
10	Milwaukee	Wisconsin		577,222	249.2	2,300	28
11	New York City	New York	Eastern Time	8,804,190	778.3	11,312	1
12	Washington	District of Columbia		689,545	158.2	4,358	2
13	Boston	Massachusetts		675,647	125.1	5,401	4
14	Philadelphia	Pennsylvania		1,603,797	348.1	4,607	8
15	Pittsburgh	Pennsylvania		302,971	143.5	2,112	9
16	Miami	Florida		442,241	93.2	4,743	10
17	Atlanta	Georgia		498,715	350.4	1,423	24
18	Phoenix	Arizona	Mountain Time	1,608,139	1,341.6	1,199	71
19	Denver	Colorado		715,522	396.5	1,805	14
20	Honolulu	Hawaii	Hawaii-Aleutian Time	350,964	156.7	2,240	13

### Data acquisition

The road network structure and travel demand are two crucial inputs for traffic assignment. In this study, we derive these data from public open-source datasets. This section provides a brief overview of the data acquisition procedures.

#### Road networks

First, the road network structures of the 20 cities are generated from the OpenStreetMap (OSM: https://www.openstreetmap.org/) database, which is an open-source mapping platform that provides crowd-sourced road network geographic information, including network topology, road attributes, and connectivity information. By leveraging OSM data, researchers gain convenient access to a comprehensive and up-to-date depiction of the network structure, which facilitates the research in urban studies^26–29^. The road attributes are also sourced from OSM. After the implementation of cleaning and integration procedures, these processed data can serve as the input for the traffic assignment. A summary of the road network data is given in Table 3.

**Table 3:** A summary of the road networks data for 20 U.S. cities.

No.	City	TAZs	Nodes	Links
1	San Francisco	194	4,986	18,002
2	Seattle	139	6,891	27,361
3	Portland	157	8,245	31,939
4	Las Vegas	175	7,823	28,831
5	Chicago	819	14,434	54,469
6	New Orleans	185	7,217	24,073
7	Austin	199	10,717	40,158
8	Minneapolis	130	4,004	15,363
9	Dallas	328	21,389	77,818
10	Milwaukee	234	8,521	30,747
11	New York City	2,005	28,626	99,410
12	Washington	179	6,136	23,573
13	Boston	191	5,542	20,487
14	Philadelphia	389	10,410	38,641
15	Pittsburgh	149	3,532	13,662
16	Miami	108	4,121	15,108
17	Atlanta	141	5,207	20,243
18	Phoenix	378	15,324	58,070
19	Denver	175	9,205	34,724
20	Honolulu	117	2,982	11,205

**Fig. 3:**
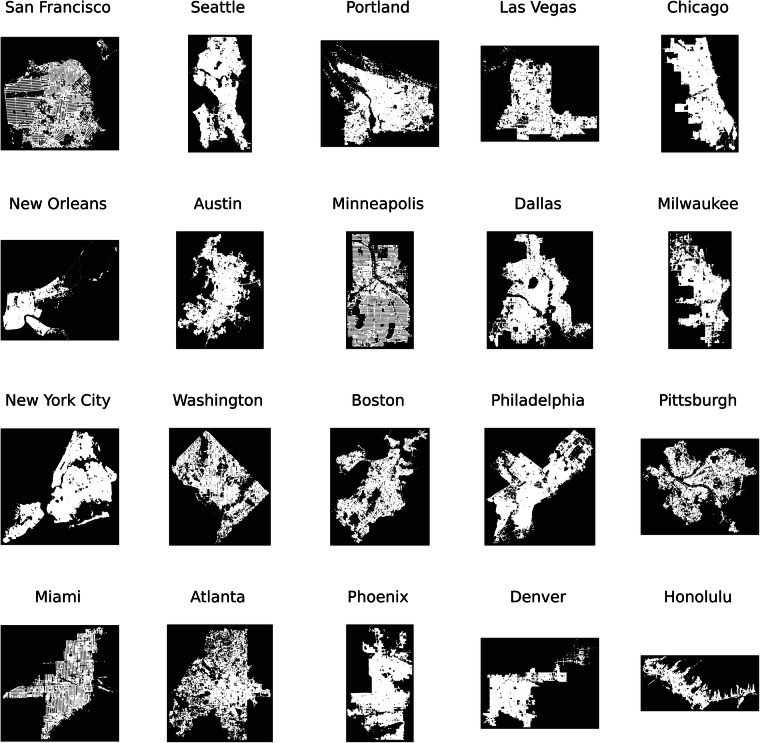
Road networks of 20 representative U.S. cities extracted from OpenStreetMap.

Specifically, we employ a Python package named osmnx^30^ (https://github.com/gboeing/osmnx) to download the OSM data. We then use another Python package called osm2gmns^31^ (https://github.com/jiawlu/OSM2GMNS) to extract the nodes and links on the road network from the OSM data and save them into separate CSV files in GMNS format^32,33^. We use five main link types including ‘motorway’, ‘trunk’, ‘primary’, ‘secondary’, and ‘tertiary’ to implement the traffic assignment. For each link type, we initiate the corresponding road attributes, including parameters such as road capacity, speed limits, the number of lanes, and so on. For the nodes, each node represents the intersection between two links and contains a unique identifier along with latitude and longitude information. By establishing the connectivity between nodes and links through their corresponding relationships, the network topology and road attributes can be constructed. We use the graphing functions of osmnx to visualize the constructed road networks of 20 representative U.S. cities in Fig. 3.

#### Travel demand

We then estimate the travel demand, another essential input data for traffic assignment, using the data from the LODES dataset (https://lehd.ces.census.gov/data/lodes/) provided by the U.S. Census Bureau. The LODES dataset includes commuting data for the workforce in all states across the United States over multiple years, which have been widely used in existing studies^34^. LODES data collection involves employers reporting employee details to state workforce agencies, including work and home locations. The U.S. Census Bureau collaborates with state agencies to process and anonymize this data. It’s then used to create Origin-Destination (OD) pairs. This dataset, at the finest granularity of block level, documents the block code for both workplace census and residence census, along with the corresponding total number of jobs. Essentially, the LODES dataset provides an excellent representation of the trip distributions of the U.S. working population that can be used to construct the OD matrix. In this study, we mainly focus on producing the traffic dataset for the year 2019 and the commuting OD data in that year are collected. Moreover, the data collection process is performed at the block level, resulting in the OD data between blocks.

#### Travel time and speed

We collect data from two open-source dataset platforms, namely TomTom (https://www.tomtom.com/traffic-index/ranking/) and Waze (https://www.waze.com/live-map/), as two indicators of travel time and average speed respectively for our dataset validation. The detailed procedures of data collection can be found in the subsequent sec:Technical ValidationTechnical Validation section.

### Data fusion

In this section, we integrate the road network data and OD data to unify the data format. Since the origins and destinations in the OD matrix are not associated with network nodes, it is infeasible to directly take these data as input for the traffic assignment. Therefore, we need to establish a connection between network nodes and blocks. After establishing the connection, we can employ the traffic assignment model to identify appropriate travel paths and allocate traffic flow to the respective links.

To be specific, we begin by aggregating the OD data from its minimum granularity at the block level to a higher level, namely, the tract level. According to the United State Bureau^35–37^, blocks are statistical units with small areas, generally defined to contain between 600 and 3,000 people, whereas tracts composed of multiple blocks are relatively larger and typically have a population size ranging from 1,200 to 8,000 people. In order to achieve a balance between computational complexity and accuracy, we consider tracts as an ideal basic unit for the traffic assignment, which is similar to the existing studies^38,39^. This implies that we use the tract as a Traffic Analysis Zone (TAZ) in the traffic assignment model.

Then, the geographical location of each TAZ is determined as the average coordinates of all the blocks within a tract. These TAZs (also called centroids) are generated and stored in the existing node file labeled with a unique identifier. Finally, we generate connectors to bridge the TAZs and network nodes. These connectors can be regarded as a special type of links that are generated from each TAZ center to their neighbor links. Moreover, these connectors are incorporated into the existing links labeled with a unique identifier. As a result, the commuting trips could start from the origin TAZ, traverse a connector to access the nearby road network, choose a suitable path, and then use another connector to reach the destination TAZ.

### Traffic assignment

In this section, we use the traffic assignment model to produce the dataset based on the User Equilibrium (UE)^40^. To be specific, we formulate the UE using an optimization model and calibrate four categories of parameters used in the model. Using the network structure and OD demand as input, the model would output the traffic flow, speed, and density on each link. Moreover, we mainly focus on the static traffic assignment and do not consider the influence of temporal variations on traffic conditions.

#### User equilibrium

All travelers naturally make decisions to minimize their own travel costs (either travel time or equivalent monetary value). Wardrop’s First Principle^41^ posits that when every traveler seeks to minimize their individual travel costs, traffic flow eventually stabilizes. In this equilibrium state, the travel costs on all utilized paths become equal and minimized. Meanwhile, the travel costs on unused paths for any given OD pair are greater than or equal to those on the used paths. In other words, a steady-state traffic condition is reached only when no traveler can improve his or her travel time by unilaterally changing routes. The satisfaction of Wardrop’s first principle is commonly referred to as User Equilibrium (UE).

The physical transport network including road segments and intersections in an urban area can be represented as a graph structure *G*(*N*, *A*) containing a link set *A* and a node set *N*. For each link *α* ∈ *A*, it has the link flow *x*_*a*_ and the link travel cost *t*_*a*_ respectively. For each node *r*, *s* ∈ *N*, it is defined as the TAZ that generates or attracts traffic demand. Therefore, the mathematical formulation of the traffic assignment model under the UE condition^42^ can be expressed as follows:

1 $$\min \;z(x)=\sum _{a}{\int }_{0}^{{x}_{a}}{t}_{a}(\omega )d\omega $$

subject to

2 $$\sum _{k}\;{f}_{k}^{rs}={q}^{rs},\quad \forall r,s;$$

3 $${x}_{a}=\sum _{r}\sum _{s}\sum _{k}\;{f}_{k}^{rs}{\delta }_{ka}^{rs},\quad \forall a;$$

4 $${f}_{k}^{rs}\ge 0,\quad \forall k,r,s,$$

where *t*_*a*_(*x*_*a*_) denotes the link performance function that indicates the travel cost on link *a* when the traffic flow is *x*_*a*_. ${f}_{k}^{rs}$ represents the traffic flow on path *k* connecting origin *r* and destination *s*. *q*^*rs*^ indicates the number of trips from origin *r* to destination *s*. ${\delta }_{ka}^{rs}$ is a binary variable indicates whether link *a* is part of path *k* between origin *r* and destination *s*. Equation (2) imposes the flow conservation constraints. Equation (3) expresses the relationship between link flow and path flow. Please refer to the book Urban Transportation Networks^40^ for details.

Once the traffic flow on each link is determined, the total travel time, denoted as ${c}_{k}^{rs}$, for a specific path *k* can be calculated by summing the travel time of each link along this path, which can be formulated as follows:

5 $${c}_{k}^{rs}=\sum _{a}{t}_{a}{\delta }_{ka}^{rs},\quad \forall k,r,s.$$

**Fig. 4:**
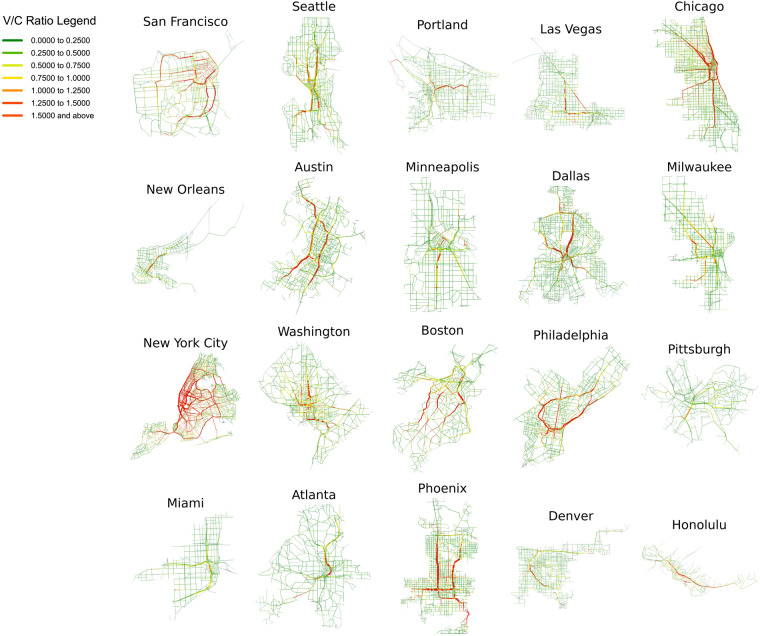
Results of the traffic assignment model in 20 representative U.S. cities.

Although the above optimization model has been proven to be a strict convex problem with a unique solution for traffic flow on links^40^, the computational cost of finding the optimal solution would significantly increase when dealing with large-scale city road networks. To alleviate the computational burden, a bi-conjugate Frank-Wolfe algorithm^43,44^ is employed to find the optimal solution. In order to enable convenient usage of the provided dataset by users from various disciplines and allow them to easily modify the core parameter settings of the traffic assignment process according to their research needs, we employ two traffic modeling platforms to generate the final dataset. Subsequent users can either directly view the dataset in a no-code format or quickly adjust parameters through a low-code approach to conduct scenario testing under different scenarios. Specifically, a commercial software (named TransCAD) and an open-source Python package for transportation modeling (named AequilibraE) are utilized simultaneously in this study. For both platforms, the maximum assignment iteration time and the convergence criteria are set to 500 and 0.001, respectively. The results of the traffic assignment model in 20 U.S. cities are shown in Fig. 4.

#### Parameters calibration

The traffic conditions on the network are influenced by many factors related to traffic supply and demand. Consequently, the traffic assignment model would be impacted and output different results. Since the disturbances in the transport system are nonlinear and challenging to quantify, it is difficult to establish a deterministic mapping relationship between various influencing factors and the results of the traffic assignment model. Therefore, we adopt a grid-search approach to calibrate four common categories of factors that are closely related to the traffic assignment model. We determine the final model by continuously fine-tuning various parameters associated with the traffic assignment model until the transport system reaches the UE condition. In this study, we introduce four categories of factors including road attributes, travel demand, impedance function, and turn penalty, as outlined below.

- Road attributes
  We categorize the entire road network into three major types, namely expressways, arterial highways, and local roads. Capacity and free flow speed of each road type are two parameters identified to be calibrated. Based on the experimental results, the appropriate range of road capacity for expressways is between 1800 veh/h/lane and 2200 veh/h/lane, while the range for free flow speed is from 65 km/h to 90 km/h. In the case of highways, the corresponding capacity value falls within the range of 1500 veh/h/lane to 2000 veh/h/lane, and the free flow speed value ranges from 40 km/h to 65 km/h. As for local roads, their capacity varies from 600 veh/h/lane to 1500 veh/h/lane, while the suitable speed ranges between 25 km/h and 45 km/h. The detailed information for each type of road can be found in Table 4.

**Table 4:** Capacity (veh/h/lane) and free flow speed (km/h) of roads.

No.	City	Capacity			Free Flow Speed		
		Expressway	Highway	Local Roads	Expressways	Highway	Local Roads
1	San Francisco	2,200	2,000	1,400	90	60	40
2	Seattle	2,200	2,000	1,400	90	65	45
3	Portland	2,200	2,000	1,400	90	65	45
4	Las Vegas	2,200	2,000	1,400	90	60	40
5	Chicago	2,000	1,900	1,400	90	60	40
6	New Orleans	2,200	2,000	1,400	90	60	40
7	Austin	2,200	2,000	1,400	90	65	40
8	Minneapolis	2,200	2,000	1,300	90	65	40
9	Dallas	2,200	2,000	1,400	90	65	45
10	Milwaukee	2,200	2,000	1,400	90	65	45
11	New York City	2,200	2,000	1,400	90	60	40
12	Washington	1,800	1,500	600	60	40	25
13	Boston	2,200	2,000	1,300	60	45	30
14	Philadelphia	2,000	1,800	1,200	90	60	30
15	Pittsburgh	2,200	2,000	1,200	90	60	30
16	Miami	1,800	1,500	900	65	50	35
17	Atlanta	2,200	2,000	1,400	70	50	35
18	Phoenix	2,200	2,000	1,500	90	65	45
19	Denver	2,000	1,800	1,300	90	60	35
20	Honolulu	2,200	2,000	1,400	90	60	40
- Travel demand
  The OD travel demand is another significant factor influencing the outcome of the traffic assignment. In this study, we aim to simulate the traffic conditions during the peak hours. As mentioned above, the OD demand matrix is derived from the total number of jobs in the United States in 2019, generated from LODES datasets. Although it is reasonable to assume that commuting travel accounts for the majority during peak hours, such demand cannot reflect the actual traffic conditions. Therefore, it is necessary to adjust the initial OD demand, considering variations in transport modes, travel departure time, and carpooling availability during commuting to work. To address this issue, we introduce an OD multiplier to estimate the actual traffic demand during the commuting time. We find that stable results can be obtained when the parameter ranges from 0.55 to 0.65. We show the travel demand and the percentage of internal travel within each TAZ in Fig. 5.

**Fig. 5:**
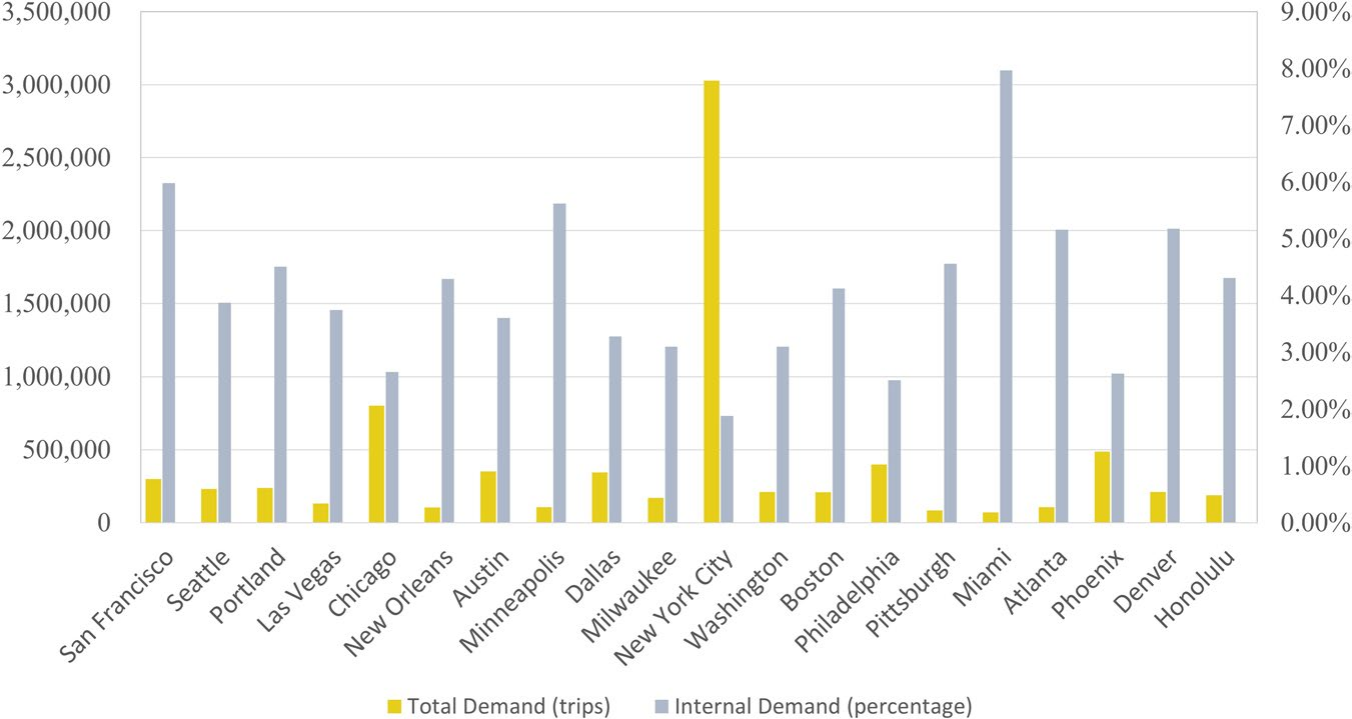
Total travel demand and the percentage of internal travel demand for 20 U.S. cities.
- Link performance function
  The link performance function, also known as the impedance function or volume delay function, refers to the relationship between travel time and traffic flow on a road. Typically, travel time increases non-linearly with the increase in traffic flow, which also significantly affects the traffic assignment. One of the most commonly adopted functions in the literature is called the Bureau of Public Roads (BPR) function^45^, which is expressed as follows:
  6 $$t={t}_{0}\left[1+\alpha {\left(\frac{v}{c}\right)}^{\beta }\right].$$
  In the function above, *t* indicates the actual travel time on the road while *t*_0_ represents the free flow travel time on the corresponding road. *v* and *c* are the traffic flow and capacity of the road, respectively. *α* and *β* are parameters needed to be fine-tuned. We find that the results are satisfactory when parameter *α* ranges from 0.15 to 0.6 while parameter *β* changes from 1.2 to 3. The specific values of parameters for each city are provided in Table 5.

**Table 5:** Parameters for BPR function and turn penalty (minutes).

No.	City	BPR Function		Turn Penalty			
		*α*	β	Left	Right	Through	U-turn
1	San Francisco	0.5	1.8	0.3	0.2	0.1	−1
2	Seattle	0.6	3	0.3	0.2	0.1	−1
3	Portland	0.5	1.2	0.2	0.15	0.1	−1
4	Las Vegas	0.5	1.3	0	0	0	−1
5	Chicago	0.5	1.2	0.3	0.2	0.1	−1
6	New Orleans	0.6	1.8	0.15	0.1	0.05	−1
7	Austin	0.5	1.5	0.3	0.2	0.1	−1
8	Minneapolis	0.15	1.8	0.15	0.1	0.05	−1
9	Dallas	0.6	1.3	0.25	0.15	0.1	−1
10	Milwaukee	0.5	1.5	0.1	0.05	0	−1
11	New York City	0.25	1.5	0.2	0.15	0.1	−1
12	Washington	0.5	1.5	0.2	0.1	0.05	−1
13	Boston	0.25	2	0.2	0.1	0.05	−1
14	Philadelphia	0.5	1.2	0.3	0.2	0.1	−1
15	Pittsburgh	0.5	2	0.35	0.25	0.15	−1
16	Miami	0.5	1.5	0.35	0.25	0.15	−1
17	Atlanta	0.2	1.5	0.25	0.15	0.1	−1
18	Phoenix	0.15	1.2	0	0	0	−1
19	Denver	0.5	1.5	0.3	0.2	0.1	−1
20	Honolulu	0.5	1.5	0.1	0.05	0	−1
- Turn penalty

The turning delay at intersections is also a significant factor that should not be dismissed. When vehicles pass through road intersections, their speed typically decreases, either due to signal control or the necessity to make turns. However, this behaviour cannot be adequately represented in solving traffic assignment problems. To ensure that the results of the traffic assignment model are in accordance with real-world scenarios, we uniformly set corresponding parameters for all junctions to simulate the turning delay effects. In other words, the turn penalty parameters are an average value for the turning delay at all intersections in the road network and these intersection types include signal-controlled intersections, roundabouts, yield or stop intersections, and others. Specifically, the time delay for right turns varies between 0 and 0.25 minutes, while the penalty for making a left turn ranges from 0 to 0.35 minutes. The delay for through traffic is between 0 and 0.15 minutes. U-turn is prohibited in the traffic assignment simulation. The specific parameter setting is demonstrated in Table 5.

## Data Records

We share the traffic dataset on a public repository (Figshare^46^). In this dataset, each folder, named after the city, contains the input and output of the traffic assignment model specific to that city. We elaborate on the details as follows:

### Input data

This folder contains all the input data required for the traffic assignment model, namely the OD demand data and network data. The network data contains both node and link files in a CSV format. The data in this file folder specifically includes the following contents:

- the initial network data obtained from OSM
- the visualization of the OSM data
- processed node/link/od data

The detailed meanings of the fields contained in different input data are given in Table 6.

**Table 6:** Fields description for the input data folder.

Data	Field	Description
Node	Node_ID	The unique identifier for each node.
	Lon	Longitude.
	Lat	Latitude.
	Tract_Node	A binary to determine whether a node point is a TAZ.
Link	Link_ID	The unique identifier for each link.
	From_Node_ID	The node ID of the starting point of this link.
	To_Node_ID	The node ID of the ending point of this link.
	Capacity	The default link capacity (veh/h).
	Length	The link length (meters).
	Free_Speed	The default link free flow speed (km/h).
	Lanes	The number of lanes.
	Link_Type	The supported link types in osm2gmns. (1 for motorway, 2 for trunk, 3 for primary, 4 for secondary, 5 for tertiary)
OD	O_ID	The node ID of the origin TAZ point.
	D_ID	The node ID of the destination TAZ point.
	OD_Number	The corresponding travel demand between the origin and destination.

### TransCAD results

This folder contains all the input data required for the traffic assignment model in TransCAD, as well as the corresponding output data. The data in this file folder specifically includes the following contents:

- cityname.dbd: geographical network database of the city supported by TransCAD
- cityname_link.shp/cityname_node.shp: network data supported by the GIS software, which can be imported into TransCAD manually
- od.mtx: OD matrix supported by TransCAD
- LinkFlows.bin/LinkFlows.csv: results of the traffic assignment model by TransCAD
- ShortestPath.mtx/ue_travel_time.csv: the travel time (in minutes) between OD pairs by TransCAD

The detailed meanings of the fields contained in output data generated from TransCAD are given in Table 7.

**Table 7:** Fields description for the TransCAD output data folder.

Data	Field	Description
Output	ID	The unique identifier generated automatically by TransCAD.
	Flow	The output assigned traffic flow (veh/h).
	Tot_Flow	The total traffic flow (veh/h) for both directions.
	Time	The loaded travel time (in minutes) for each link.
	Max_Time	The maximum loaded travel time (in minutes) for links in both directions.
	VOC	The Volume to Capacity (V/C) ratio for each link.
	Max_VOC	The maximum V/C ratio for links in both directions.
	VKmT	The Vehicle-Kilometre Travelled for each link.
	Tot_VKmT	The total Vehicle-Kilometre Travelled for both directions.
	VHT	The Vehicle-Hours Travelled for each link.
	Tot_VHT	The total Vehicle-Hours Travelled for both directions.
	Speed	The loaded travel speed (km/h) for each link.
	VDF	The loaded cost (result from the Volume Delay Function) for each link.
	Max_VDF	The maximum loaded cost for links in both directions.

### AequilibraE results

This folder contains all the input data required for the traffic assignment model in AequilibraE, as well as the corresponding output data. The data in this file folder specifically includes the following contents:

- cityname.shp: shapefile network data of the city support by QGIS or other GIS software
- od_demand.aem: OD matrix supported by AequilibraE
- network.csv: the network file used for traffic assignment in AequilibraE
- assignment_result.csv: results of the traffic assignment model by AequilibraE

The detailed meanings of the fields contained in output data generated from AequilibraE are given in Table 8.

**Table 8:** Fields description for the AequilibraE output data folder.

Data	Field	Description
Output	link_id	The unique identifier generated automatically by AequilibraE.
	matrix_ab	The output assigned traffic flow (veh/h) for the AB direction.
	matrix_ba	The output assigned traffic flow (veh/h) for the BA direction.
	matrix_tot	The total output assigned traffic flow (veh/h) for both directions.
	Congested_Time_AB	The congested link travel time for the AB direction.
	Congested_Time_BA	The congested link travel time for the BA direction.
	Congested_Time_Max	The maximum congested link travel time for both directions.
	Delay_factor_AB	The ratio of congested travel time to free flow travel time for the AB direction.
	Delay_factor_BA	The ratio of congested travel time to free flow travel time for the BA direction.
	Delay_factor_Max	The maximum ratio of congested travel time to free flow travel time for both directions.
	VOC_AB	The Volume to Capacity (V/C) ratio for the AB direction.
	VOC_BA	The Volume to Capacity (V/C) ratio for the BA direction.
	VOC_max	The maximum V/C ratio for both directions.
	PCE_AB	The output assigned traffic flow (PCE/h) for the AB direction.
	PCE_BA	The output assigned traffic flow (PCE/h) for the BA direction.
	PCE_tot	The total output assigned traffic flow (PCE/h) for both directions.

## Technical Validation

To ensure the consistency between the traffic assignment model’s output and real-world traffic conditions, we conduct validation using two different public open sources of traffic data. Specifically, the travel time between different OD pairs and the overall average travel speed are employed as two validation indicators to ensure the reliability and accuracy of the provided dataset. The validation results are shown in Tables 9, 10 and we can see that the provided dataset for each city is accurate and valid.

**Table 9:** Correlation analysis for the average travel time (min) among Waze, TransCAD and AequilibraE.

No.	City	Traffic Assignment Results				Correlation Coefficient (*R*^2^)	
		UETT	FFTT	Delay	Delay Factor	Waze vs. TransCAD	TransCAD vs. AequilibraE
1	San Francisco	17.60	13.09	4.51	1.34	0.83	0.86
2	Seattle	19.43	15.74	3.69	1.23	0.70	0.78
3	Portland	18.14	14.82	3.31	1.22	0.72	0.94
4	Las Vegas	16.19	12.95	3.25	1.25	0.83	0.96
5	Chicago	27.48	16.68	10.80	1.65	0.86	0.94
6	New Orleans	14.44	12.15	2.29	1.19	0.89	0.96
7	Austin	23.69	18.80	4.89	1.26	0.82	0.96
8	Minneapolis	10.25	9.78	0.47	1.05	0.75	0.97
9	Dallas	26.16	20.20	5.96	1.30	0.79	0.93
10	Milwaukee	12.87	10.60	2.26	1.21	0.78	0.97
11	New York City	44.14	19.67	24.47	2.24	0.88	0.80
12	Washington	17.32	13.83	3.49	1.25	0.75	0.91
13	Boston	17.50	14.80	2.70	1.18	0.77	0.93
14	Philadelphia	22.94	17.43	5.51	1.32	0.85	0.92
15	Pittsburgh	15.62	14.82	0.80	1.05	0.72	0.92
16	Miami	14.20	13.22	0.98	1.07	0.73	0.93
17	Atlanta	17.85	16.91	0.93	1.06	0.72	0.98
18	Phoenix	20.32	16.68	3.64	1.22	0.77	0.93
19	Denver	19.76	17.47	2.28	1.13	0.78	0.97
20	Honolulu	12.84	8.70	4.14	1.48	0.86	0.94
**Average**						**0.79**	**0.92**

**Table 10:** Comparison of the average speed (km/h) generated from our model and TomTom Traffic Index.

No.	City	Traffic Assignment Results				Comparison Results				
		Total VKMT	Total VHT	Link-based Speed	OD-based Speed	TomTom Speed	Link-based MAPE	Link-based MAE	OD-based MAPE	OD-based MAE
1	San Francisco	1,064,455	38,297	27.79	25.33	25	11.18%	2.79	1.32%	0.33
2	Seattle	1,122,897	32,232	34.84	27.10	35	0.46%	0.16	22.57%	7.90
3	Portland	1,350,385	33,513	40.29	34.40	37	8.90%	3.29	7.03%	2.60
4	Las Vegas	994,459	21,477	46.30	37.39	43	7.68%	3.30	13.05%	5.61
5	Chicago	5,219,394	178,194	29.29	29.68	27	8.48%	2.29	9.93%	2.68
6	New Orleans	519,398	12,552	41.38	36.79	38	8.89%	3.38	3.18%	1.21
7	Austin	2,869,374	68,516	41.88	36.47	38	10.21%	3.88	4.03%	1.53
8	Minneapolis	395,714	8,842	44.75	34.22	43	4.08%	1.75	20.42%	8.78
9	Dallas	3,110,055	73,012	42.60	36.02	40	6.49%	2.60	9.95%	3.98
10	Milwaukee	940,291	21,068	44.63	38.73	44	1.44%	0.63	11.98%	5.27
11	New York City	22,955,902	1,112,278	20.64	23.02	20	3.19%	0.64	15.10%	3.02
12	Washington	761,414	30,147	25.26	21.90	24	5.24%	1.26	8.75%	2.10
13	Boston	791,389	30,018	26.36	23.94	26	1.40%	0.36	7.92%	2.06
14	Philadelphia	2,298,316	67,212	34.19	30.05	32	6.86%	2.19	6.09%	1.95
15	Pittsburgh	325,372	8,883	36.63	31.51	36	1.74%	0.63	12.47%	4.49
16	Miami	244,779	7,196	34.02	32.35	34	0.05%	0.02	4.85%	1.65
17	Atlanta	596,929	14,961	39.90	32.61	39	2.30%	0.90	16.38%	6.39
18	Phoenix	5,152,506	99,152	51.97	43.67	53	1.95%	1.03	17.60%	9.33
19	Denver	1,222,654	32,359	37.78	32.14	35	7.95%	2.78	8.17%	2.86
20	Honolulu	779,801	23,275	33.50	29.27	32	4.70%	1.50	8.53%	2.73
**Average**							**5.16%**	**1.77**	**10.47%**	**3.82**

### Travel time

In examining the travel time metric, we obtain the travel time between different OD pairs both from traffic assignment models and map service providers. As for the model side, the travel time under both UE and free flow conditions are calculated respectively using traffic assignment models. First, under UE conditions, the travel time between different OD pairs could be generated by summing the link travel time determined by the corresponding assigned traffic flow along the shortest path as shown in Eq. (5). Then, under free flow conditions, the travel time between OD pairs is the travel time associated with the shortest path, disregarding congestion on road segments. Furthermore, the average value of Travel Time (in minutes) under UE conditions (UETT) as well as free flow conditions (FFTT) for all OD pairs can be expressed as follows:

7 $$UETT=\frac{\mathop{\sum }\limits_{r}^{N}\mathop{\sum }\limits_{s}^{N}{c}_{ue}^{rs}{q}^{rs}}{\mathop{\sum }\limits_{r}^{N}\mathop{\sum }\limits_{s}^{N}{q}^{rs}};$$

8 $$FFTT=\frac{\mathop{\sum }\limits_{r}^{N}\mathop{\sum }\limits_{s}^{N}{c}_{ff}^{rs}{q}^{rs}}{\mathop{\sum }\limits_{r}^{N}\mathop{\sum }\limits_{s}^{N}{q}^{rs}},$$

where ${c}_{ue}^{rs}$ and ${c}_{ff}^{rs}$ denote the travel time between origin *r* and destination *s* under the UE and free flow conditions respectively. Additionally, the difference as well as the ratio between these two types of travel time give the average travel delay (in minutes) and delay factor for each city.

In terms of the real-world data for validation, since nowadays many map service providers have the capability to offer travel time estimates between two location points at different departure times based on users’ historical navigation records, in this study, we choose Waze as the data source to obtain the actual travel time between each OD pair by using its WazeRouteCalculator API (https://github.com/kovacsbalu/WazeRouteCalculator) with Python code.

The results of travel time are shown in Table 9. It can be seen that Honolulu experiences the least travel time under free flow conditions, at about 8.70 minutes, while Minneapolis has the shortest average travel time during commuting hours, at about 10.25 minutes. Minneapolis also has the lowest delay travel time among all cities, merely 0.47 minutes, indicating that the commuting travel time in this city is almost the same as the travel time under free flow conditions. In contrast, New York City experiences significant delays, with a delay time of 24.47 minutes, revealing that the travel time during peak periods in New York is more than double that of the free flow condition. In terms of the delay factor, New York City has the highest value, reaching 2.24, followed by Chicago with a value of 1.65. Minneapolis and Pittsburgh have the lowest delay factor values, both at 1.05.

To evaluate the results, we use the Pearson Correlation Coefficient (PCC)^47^ to measure the correlation between the actual travel time and the travel time produced by our model. The PCC *r*_*xy*_ is defined as follows:

9 $${r}_{xy}=\frac{n\sum {x}_{i}{y}_{i}-\sum {x}_{i}\sum {y}_{i}}{\sqrt{n\sum {x}_{i}^{2}-(\sum {x}_{i}{)}^{2}}\sqrt{n\sum {y}_{i}^{2}-{(\sum {y}_{i})}^{2}}},$$

where *r*_*xy*_ denotes the Pearson’s Correlation Coefficient. *x*_*i*_ and *y*_*i*_ are the individual sample points indexed with *i*. *n* represents the sample size.

Since the turning penalties are not incorporated in the traffic assignment algorithm of AequilibraE, the parameter settings in TransCAD and AequilibraE are not identical. Consequently, results of the two platforms are not entirely consistent. Considering the more comprehensive parameter settings in TransCAD, we adopt the results of TransCAD as the primary benchmark. We perform PCC analysis between Waze and TransCAD, as well as between TransCAD and AequilibraE, with the evaluation results presented in Table 9.

**Fig. 6:**
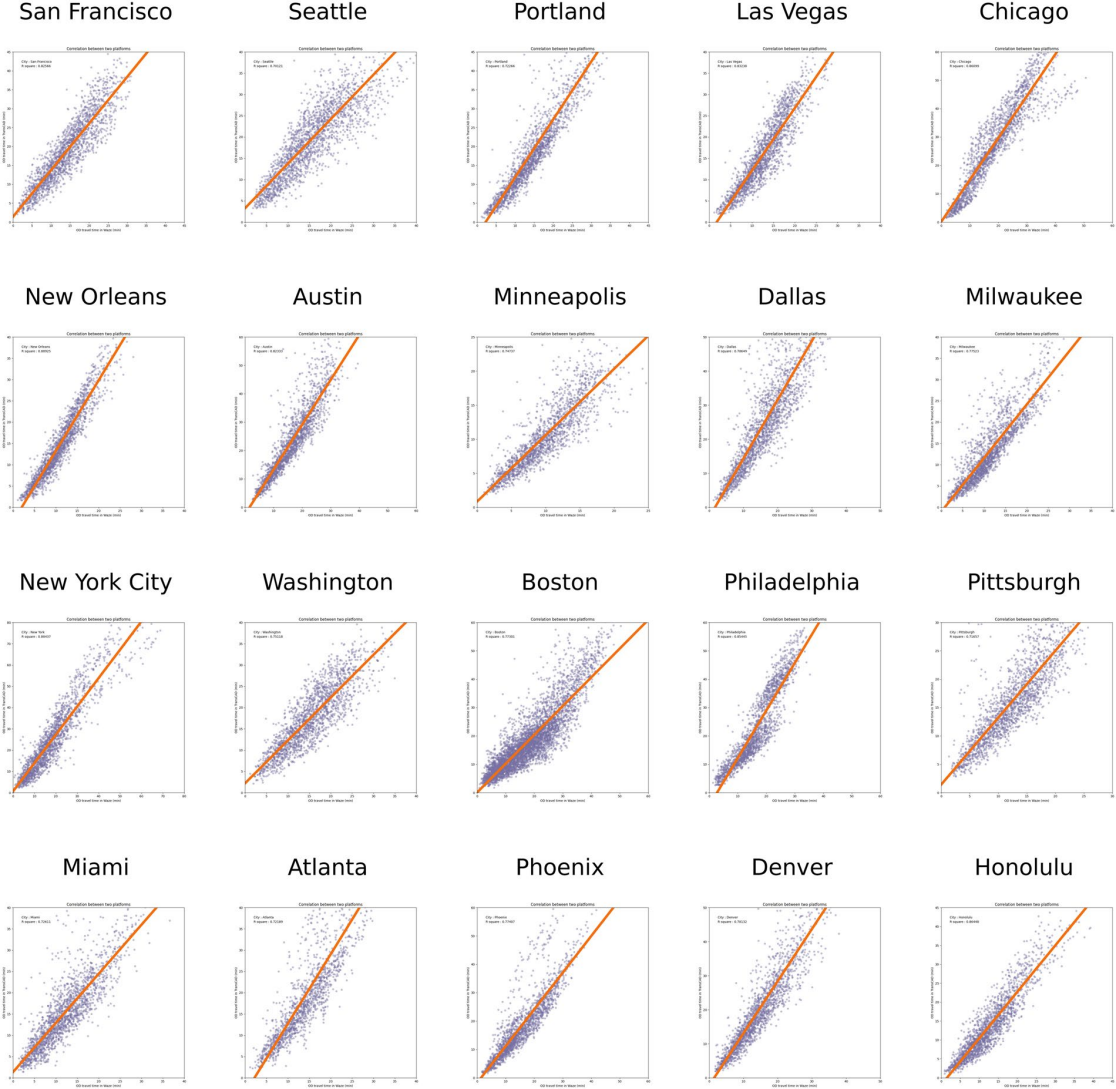
Correlation analysis results between Waze and TransCAD.

From the correlation analysis, we can find that all correlation coefficients *R*^2^ are greater than 0.7, which confirms the accuracy and reliability of the results to some extent. We also visualize the correlation coefficient for each city in Fig. 6. It can be seen that the simulated travel time is consistent with the travel time in the real world.

### Average speed

The overall average speed of the entire road network is another important indicator for validation. In this study, we use the speed data collected from TomTom Traffic Index as the actual speed to validate our model. We first calculate the average link-based speed of our model through dividing Vehicle Hours Travelled (VHT) by Vehicle Kilometers Travelled (VKMT). Then, the average OD-based speed values are derived from the ratio of distance to travel time between each OD pair. The Mean Absolute Percent Errors (MAPE) and Mean Absolute Errors (MAE) for both the link-based speed and the OD-based speed are used to measure the reliability of our model:

10 $$MAPE=\frac{1}{n}\mathop{\sum }\limits_{i=1}^{n}\left|\frac{{y}_{i}-{\widehat{y}}_{i}}{{y}_{i}}\right|;$$

11 $$MAE=\frac{1}{n}\mathop{\sum }\limits_{i=1}^{n}\left|{y}_{i}-{\widehat{y}}_{i}\right|,$$

where *y*_*i*_ is the actual observed value, ${\widehat{y}}_{i}$ is the predicted value, and *n* is the number of samples.

The results are summarized in Table 10. We find that the average MAPE and MAE values for the link-based speed metric are 5.16% and 1.77 km/h, respectively. Moreover, the average MAPE and MAE values for the OD-based speed indicator are 10.47% and 3.82 km/h, respectively. This implies that our approach can produce satisfactory and reliable results.

### Network traffic impact on model performance

To validate the effectiveness and robustness of our model across cities, we further investigate how traffic conditions of a city affect the model performance. The MAE and MAPE values for link-based average speed metrics obtained in Table 10 are used to evaluate the model performance. The traffic conditions are characterized by two different indicators. One is the ratio of the total OD travel demand to the number of links for the entire road network, which can characterize the average OD demand and represent the traffic conditions of a city. The other is the average speed (km/h) in rush hour obtained from TomTom (refer to Table 10). If the values of average traffic demand are large, it reveals a congested city network experiencing substantial traffic demand, exemplified by cities like New York and San Francisco. Conversely, a small value suggests a city road network with low traffic demand, as observed in cities like Atlanta and Dallas. We can draw similar conclusions with respect to the average traffic speed.

**Fig. 7:**
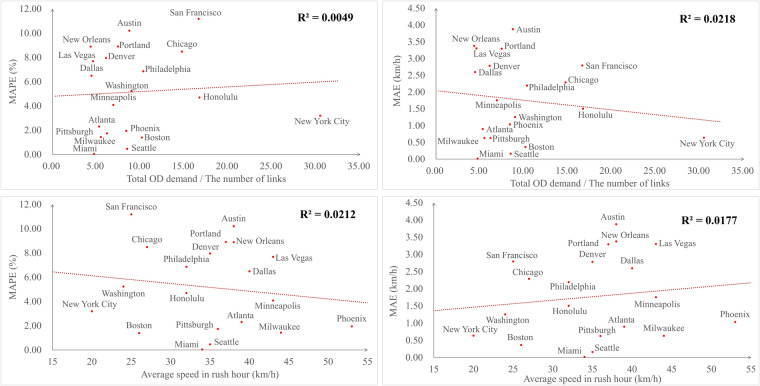
The model performance in relation to different traffic conditions for 20 U.S. cities. (**a**) The MAPE values (%) regarding the average OD demand for different cities. (**b**) The MAE values (km/h) regarding to the average OD demand for different cities. (**c**) The MAPE values (%) regarding the average speed for different cities. (**d**) The MAE values (km/h) regarding the average speed for different cities.

The results are shown in Fig. 7. The red dashed line represents the linear regression trendline that has been fitted to the data points. The *R*^2^ values of Fig. 7a and Fig. 7b are 0.0049 and 0.0218, respectively. This implies that there is no evident relationship between the model performance and the varying traffic demand of the network. Similarly, the *R*^2^ values of Fig. 7c and Fig. 7d are 0.0212 and 0.0177, respectively. This suggests that the model performance is not affected by the varying traffic speeds in different cities. In summary, the proposed model exhibits low sensitivity to variations in city traffic conditions and achieves satisfactory performance across cities.

## Usage Notes

The acquisition of OD data is crucial in performing the traffic assignment and producing the citywide traffic dataset. In this study, we utilize the commuting OD data (LODES) provided by the U.S. Census Bureau to generate the OD matrix. For cities in other countries, OD data can be substituted with alternative open data sources, such as OD data provided by TomTom (https://developer.tomtom.com/od-analysis/documentation/product-information/introduction).

Moreover, we use the average traffic time and average travel speed between different OD pairs in the real world to validate the results of our approach, ensuring its reliability and accuracy. If additional data sources are available, such as traffic flow data obtained from traffic detectors, we can also use the corresponding data to further evaluate the effectiveness of the provided dataset.

It is worth noting that the provided dataset is mainly used for macroscopic urban research and policy development across interdisciplinary studies. In view of this, the given dataset provides full spatial coverage of the entire road network, unlike existing traffic datasets that focus on specific areas. Hence, the provided traffic dataset and existing traffic datasets complement each other, which can better facilitate research in urban studies. Specifically, the full spatial coverage of the provided dataset makes it valuable for comprehensive macroscopic urban research and policy development, making a notable contribution to the literature, such as public transport planning, road expansions, the determination of bus routes, the estimation of the transport-related environmental impact and so on. In contrast, existing traffic datasets (e.g., PeMS) may exhibit incomplete spatial coverage, making them less suitable for the aforementioned macroscopic urban studies. Actually, the datasets containing fine-grained temporal information are more suitable for investigating regional traffic dynamics by leveraging the spatiotemporal relationship between the traffic data, such as traffic prediction, spatiotemporal propagation of shockwaves, calibration of fundamental diagrams, traffic data imputation, and so on.

In this study, the provided dataset lacks fine-grained temporal information due to the limited availability of input data. To fully understand dynamic traffic patterns, it is essential to consider both spatial and temporal dimensions within the traffic data. Consequently, developing a dynamic traffic assignment model that effectively captures the spatiotemporal interdependencies of traffic data is important. Moreover, employing daily traffic data for more fine-grained validation would enhance further urban research.

## Data Availability

The guidelines for data retrieval and utilization have been uploaded to GitHub^48^. The specific contents comprise: 1. **Input data introduction.ipynb**: A brief introduction and data demonstration about the input data for the traffic assignment process in the dataset. 2. **A guide for TransCAD users.md**: It is a guide for users who want to view and modify the dataset in the Graphical User Interface (GUI) of TransCAD. 3. **AequilibraE_assignmnet.py**: A Python code file for users who want to get access to the traffic assignment results by using the AqeuilibraE.
